# Correlation of Molecular Markers, *Pfmdr1*-N86Y and *Pfcrt*-K76T, with *In Vitro* Chloroquine Resistant *Plasmodium falciparum*, Isolated in the Malaria Endemic States of Assam and Arunachal Pradesh, Northeast India

**DOI:** 10.1371/journal.pone.0103848

**Published:** 2014-08-08

**Authors:** Sandeep Kumar Shrivastava, Ravi Kumar Gupta, Jagdish Mahanta, Mohan Lal Dubey

**Affiliations:** 1 Department of Parasitology, Post Graduate Institute of Medical Education and Research, Chandigarh, India; 2 Basic and Clinical Immunology of Parasitic Diseases, Centre for Infection and Immunity of Lille, Institut Pasteur de Lille, France; 3 Central Research Institute, Kasauli (H.P.) India; 4 Regional Medical Research Centre (ICMR), Dibrugarh, Assam, India; Swiss Tropical & Public Health Institute, Switzerland

## Abstract

The mechanism of chloroquine (CQ) resistance in *Plasmodium falciparum* is not clearly understood. However, CQ resistance has been shown to be associated with point mutations in *Pfcrt* and *Pfmdr1*. These genes encode for digestive vacuole transmembrane proteins Pfcrt and Pgh1, respectively. The present study was carried out to analyze the association of *Pfcrt*-K76T and *Pfmdr1*-N86Y mutations with CQ resistance in Northeast Indian *P. falciparum* isolates. 115 *P. falciparum* isolates were subjected to *in vitro* CQ sensitivity testing and PCR-RFLP analysis for the *Pfmdr1*-N86Y and *Pfcrt*-K76T mutations. 100 isolates of *P. falciparum* were found to be resistant to CQ by the *in vitro* susceptibility test (geometric mean EC_50_ 2.21 µM/L blood) while 15 were found to be CQ sensitive (geometric mean EC_50_ 0.32 µM/L blood). All the CQ resistant isolates showed the presence of *Pfmdr1* and *Pfcrt* mutations. CQ sensitive isolates were negative for these mutations. Strong linkage disequilibrium was observed between the alleles at these two loci (*Pfmdr1-*N86Y and *Pfcrt-*K76T). The results indicate that *Pfmdr1*-N86Y and *Pfcrt*-K76T mutations can be used as molecular markers to identify CQ resistance in *P. falciparum.* The result necessitates the evaluation of CQ *in vivo* therapeutic efficacy in endemic areas for more effective malaria control strategies.

## Introduction

Malaria is one of the major public health problems of the malaria affected countries, including India. In India, around 1.5 million laboratory confirmed cases of malaria are reported annually, out of which 50% cases are due to *Plasmodium falciparum* alone. Chloroquine (CQ) has been the most effective drug in the treatment of non-complicated malaria. A sudden rise in *P. falciparum* cases has been caused by resistance towards CQ, which was used for a long time as the first line of treatment of malaria cases [1]. CQ resistance may lead to high morbidity and mortality in *P. falciparum* cases, if not treated timely. CQ acts by interfering with heme metabolism in the digestive vacuole of *P. falciparum* and CQ resistance results from reduced accumulation of the drug by the parasites [2]–[4].

Various genetic alterations have been shown to be associated with CQ resistance. Mainly, two genes known as *P. falciparum* multidrug resistance gene *Pfmdr1*, which codes for Pgh1, a P-glycoprotein homologue, and the CQ resistance transporter gene *Pfcrt*, which codes for CQ resistance transporter protein have been identified as potential candidates of CQ resistance. Several point mutations in *Pfmdr1* gene at positions 754, 1049, 3598, 3622 and 4234 result in amino acid changes at codons 86, 184, 1034, 1042 & 1246, respectively. These amino acid changes have been shown to be associated with CQ resistance [5]–[12]. Out of the several mutations described, the mutation in codon 86 (from asparagine to tyrosine, N86Y), involved in the substrate specificity of the gene product (P- glycoprotein), appears to be the most important as this may alter the transport activity of the protein [4]. However, a few studies have reported contrasting observations with regard to the role of *Pfmdr1* gene mutations in CQ resistance [13]. Southeast Asian CQ resistant isolates (K1 genotype) have shown N86Y mutation while CQ resistant South American isolates (7G8 genotype) were negative for N86Y, and showed mutations at positions 184, 1034, 1042 and 1246 [5]. Mutation in codon 86 has also been correlated to CQ resistance in parasites selected *in vitro* for CQ resistance [13]. Similarly, mutations in the *Pfcrt* (codon 74, 75, 76, 220, 271, 326, 371) have also been shown to play a role in *in vitro* CQ resistance in laboratory lines of *P. falciparum* from all over the world [14]–[16]. *Pfcrt* K76T mutation has not been observed in CQ responders, and therefore, has been accepted as a good molecular marker for CQ resistance in *P. falciparum* [10], [17]–[19].

Malaria is a serious health problem in India, especially in the Northeast. However, to date, the role of mutations in genes *Pfmdr1* and *Pfcrt* has not been studied in the emergence of *P. falciparum* CQ resistance. Although studies from other parts of India have reported poor association of CQ resistance with these gene mutations, but no extensive study has been carried out, yet [20]. In *P. falciparum* endemic areas, CQ was the recommended first line treatment for uncomplicated malaria. However, now a days this has been changed to artesunate-based combination therapies. Despite this, in many malaria-affected areas CQ is still used for non-complicated malaria [11], [21]. Therefore, constant observation of the existing parasite population genetic makeup and determination of the presence of CQ resistance is important [22]. Keeping this in mind, the present study was planned to explore the correlation between *in vitro* CQ sensitivity and *Pfcrt*-K76T and *Pfmdr1*-N86Y mutations in a large number of clinical isolates of *P. falciparum* from the Northeast India. Since fresh isolates often have a mixture of clones of both CQ sensitive and resistant clones, the culture-adapted line derived from an isolate often responds differently. Therefore, in the present study, fresh clinical isolates were used. The linkage disequilibrium between the alleles in codon 86 of *Pfmdr1* (N86 & 86Y) and in codon 76 of *Pfcrt* (K76 & 76T) gene were also analyzed.

## Materials and Methods

### Ethics Statement

Ethical Committee of Post Graduate Institute of Medical Education & Research, Chandigarh approved the study protocol. The institutional ethics committee adhered to guidelines of national regulatory agency i.e. ICMR for conductance of experiments on humans and animals.

### Study area and sample collection

This study was carried out in the remote villages of (Tinsukhia, Assam and Lohit, Arunachal Pradesh) of Northeast India. These area's have big tea estates and heavy rainfall throughout the year, and hence malaria too. The study participants were poor tea estate workers, lacking ready access to medical services. Informed written consent was obtained from the patients. For drug sensitivity and molecular analysis, approximately 5 ml of blood was collected from the asymptomatic patients (25–45 years old) who were tested positive for *P. falciparum* using Giemsa staining. The blood was stored in vials containing citrate and stored in at 2^o^–8°C. A total of 115 blood samples were included in the study, with 65 from Assam and 50 from Arunachal Pradesh. Samples with multiple infections were excluded from the study. The present study was carried out as a part of PhD work of Mr. Sandeep K. Shrivastava and data generated during this study was submitted to the Institute Academics Committee, and can also be accessed from the institute central library after due permission from authorities.

### *In vitro* sensitivity testing

An *in vitro* micro test (Mark III) protocol recommended by WHO [23] was followed for the sensitivity testing. The CQ sensitivity test was performed immediately after the collection of blood. The test was considered valid and interpretable if ≥10 percent of the parasites in the control well (drug free well) had developed into the schizonts after 24–36 hours incubation. Isolates were considered resistant if they showed schizont maturation at CQ concentrations ≥ 8 pmol/well (1.6 µmol/L blood). To evaluate the drug-parasite response, the EC50 value (50% inhibition) was calculated by Probit analysis.

### DNA isolation

DNA was isolated according to method of Foley et al. [24] with slight modifications. Briefly, the cells from 100 µl whole blood were lysed with 1 ml of ice-cold 5 mM Na_2_HPO_4_ (pH 8.0) and the pellet was collected by centrifugation at 10000 X g for 10 minutes. This step was repeated two times more. Finally, the pellet obtained was re-suspended in 50 µl of sterile, double distilled water. The re-suspended pellet was heated in a boiling water bath for 10 minutes, followed by cooling to room temperature and centrifugation as above. For the PCR analysis, 3 µl of the supernatant was used as DNA template.

### Detection of N86Y mutation in *Pfmdr1* gene

Nested PCR, as reported previously was performed to amplify codon 86 of *Pfmdr1* [21]. During nest1 reaction, primers P1- 5′ATGGGTAAAGAGCAGAAAGA3′ and P2-5′AACGCAAGTAATACATAAAGTCA3′ were used to amplify the region flanking codon 86. Nested primers P3 5′TGGTAACCTCAGTATCAAAGAA3′ and P4 5′ATAAACCTAAAAAGGAACTGG3′ were used to amplify the PCR product in nest2 reaction. In nest 1, PCR parameters were, initial denaturation at 94°C for 3 minutes, followed by 45 cycles, each of 30 sec at 92°C, 45 sec at 48°C, 1 min at 65°C followed by the final extension at 65°C for 5 min. In nest 2, only 20 cycles of PCR were run (Mastercycler, Eppendorf, USA).

### Restriction Digestion with ApoI and Afl III

The finally amplified product was subjected to restriction digestion with Afl III (mutational allele) and Apo I (wild type allele) (New England Biolabs, UK) by incubating at 37°C for one hour with the one unit of each enzyme. The digests were resolved on 3% agarose gel, stained with ethidium bromide, and results were recorded on the gel documentation system (UVITEC, UK).

### Detection of the K76T mutation in *Pfcrt* gene

For the K76T mutation, during nest1, primers CRTP1 5′ CCGTTAATAATAAATACACGCAG3′ and CRTP2 5′GCATGTTACAAAACTATA GTTACC3′ were used, and for nest2 CRTD1: 5′TGTGCTCATGTGTTTAAACTT3′ and CRTD2: 5′CAAAACTATAGTTACCAATTTT3′ were used [25]. The nest1 PCR parameters were, initial denaturation at 95°C for 5 minutes followed by 45 cycles, each of 30 sec at 92°C, 56 sec at 30°C, 1 min at 60°C followed by the final extension at 60°C for 3 min. In nest2 PCR, initial denaturation at 95°C for 5 minutes followed by 25 cycles, each of 30 sec at 92°C, 30 sec at 48°C, 30 sec at 65°C followed by the final extension at 65°C for 3 min were done. The nested PCR product was digested with *ApoI* as described above.

### Statistical analysis

All the experiments were repeated thrice to validate the reproducibility. The results were expressed as geometric means and were reported with 95% confidence intervals. For statistical analysis (Chi squared test), SPSS 10 and Epi-Info 6.04 (CDC, Atlanta, GA, USA) were used. Probit analysis was used to calculate the CQ EC_50_ for each isolate [22], [26]. Linkage disequilibrium constants (D' and r^2^) were also calculated [27]–[28].

## Results

A total of 115 *P. falciparum* isolates were used in the study. The *in vitro* CQ susceptibility of these isolates was carried out according to WHO guidelines and isolates were categorized as sensitive and resistant strains. Out of 65 isolates from Assam, 50 were found to be CQ resistant in the *in vitro* sensitivity test with the geometric mean EC_50_ value of 1.06 µmol/L blood (Chi^2^ value 68.44, P<10^−5^ with 95% confidence intervals), and 15 showed the sensitivity towards CQ with geometric mean EC_50_ of 0.32 µmol/L blood (Chi^2^ value 161.07, P<10^−5^ with 95% confidence intervals). On the other hand, all 50 isolates collected from Arunachal Pradesh also showed a high level of CQ resistance with geometric mean EC_50_ of 2.94 µmol/L blood (the chi^2^ or p value could not be computed due to uniformity of one parameter). In total, 100 *P. falciparum* isolates were found to be the CQ resistant (geometric mean EC_50_ 2.21 µmol/L blood) and 15 isolates were found to be the CQ sensitive (geometric mean EC_50_ 0.32 µmol/L blood) (Chi^2^ value 161.07, P<10^−5^ with 95% confidence intervals).

**Figure 1:**
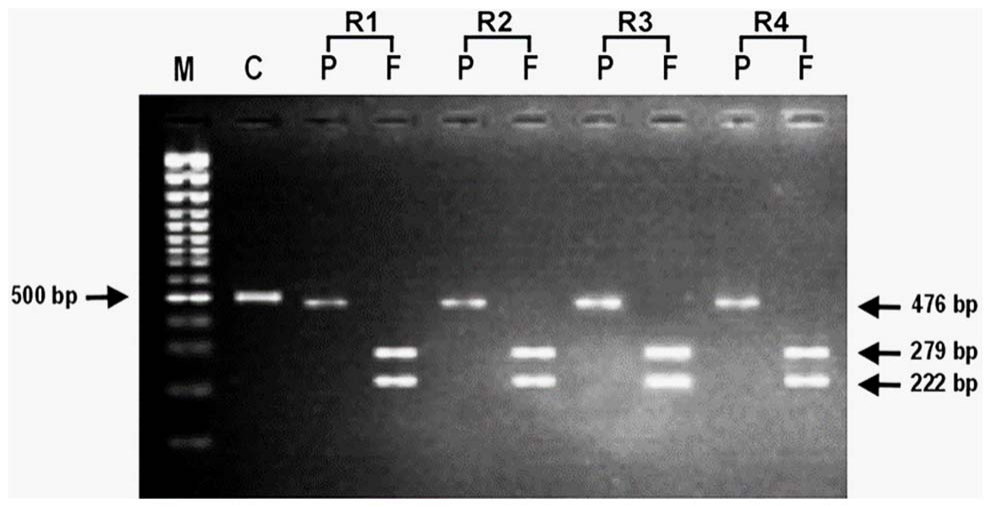
Representative photomicrograph showing the results of RFLP after digestion of PCR amplified product with endonucleases, Apo1 and AfI III for *Pfmdr1* gene (86Y) in chloroquine resistant *Plasmodium falciparum* isolates. In photograph, (M) is 100 bp DNA ladder; (C) is undigested product as control; (P) is Apo1; (F) is Afl III and (R1- R4) are chloroquine resistant *P. falciparum* isolates.

**Figure 2:**
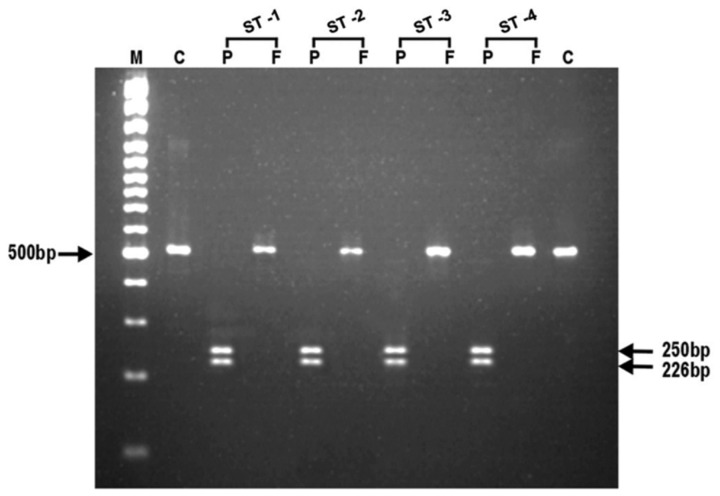
Representative photomicrograph showing the results of RFLP after digestion of PCR amplified product with endonucleases, Apo1 and AfI III for *Pfmdr1* gene (N86) in chloroquine sensitive *Plasmodium falciparum* isolates. In photograph, (M) is 100 bp DNA ladder; (C) is undigested product as control; (P) is Apo1; (F) is Afl III and (ST1-ST4) are chloroquine sensitive *P. falciparum* isolates.

**Figure 3:**
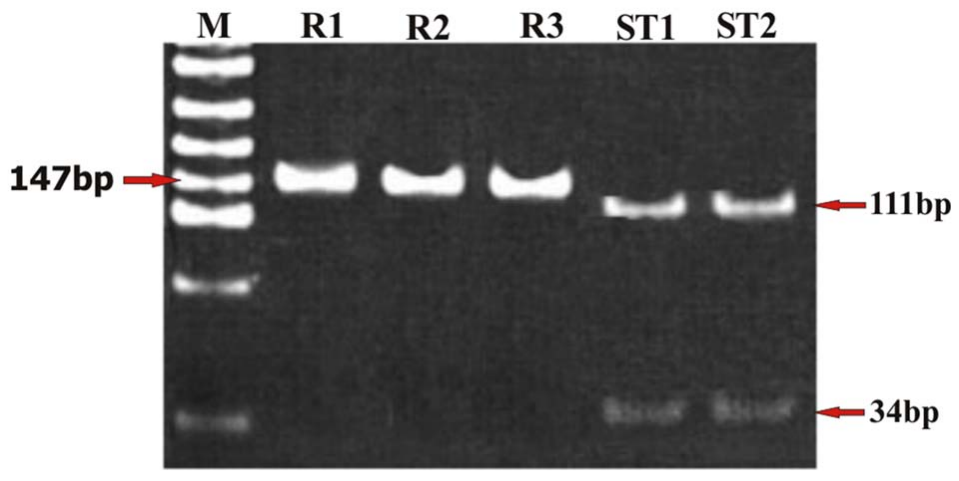
Representative photomicrograph showing the results of RFLP after digestion of PCR amplified product with endonuclease, Apo1 for *Pcfrt* gene (K76T) in chloroquine resistant and sensitive *Plasmodium falciparum* isolates. In photograph, (M) is molecular weight marker pUC mix8; (R1-R3) are chloroquine resistant *P. falciparum* isolates and (ST1-ST2) are chloroquine sensitive *P. falciparum* isolates.

To ascertain the genetic polymorphism leading to the CQ resistance, nested PCR for *Pfmdr1* and *Pfcrt* genes was performed. To confirm the status of the amplicon, the PCR product was digested with Apo1 and Afl III. On nested PCR for *Pfmdr1*, all the isolates showed the *Pfmdr1-*codon 86 region amplicon with the product size of 501 bp. On digestion with the ApoI in the case of CQ sensitive isolates, ApoI digested the amplicon at codon 86, and generated three fragments of 250 bp, 226 bp and 25 bp, respectively. Afl III was unable to digest the amplicon of the sensitive isolates (Table 1; Figure 1). However, Afl III generated two fragments of 279 bp and 222 bp, respectively from the amplicon of all the CQ resistant isolates indicating a mutant allele at codon 86 (86T). ApoI did not digest the amplicon of the CQ resistant strains at this site, and generated fragments of 476 bp and 25 bp (Table 1; Figure 2). The nested PCR of *Pfcrt* gene showed an amplicon of 145 bp. In the case of CQ sensitive isolates, the ApoI digestion of amplicon resulted in two fragments of 111 bp and 34 bp respectively, suggesting the presence of K76 allele at codon 76. On the other hand, mutant allele 76T was observed in all the CQ resistant isolates with an undigested fragment of 145 bp (Table 1; Figure 3).

**Table 1:** Chloroquine sensitivity status and presence of *pfmdr1*-N86Y and *pfcrt*-K76T mutation in *P. falciparum* isolates from Northeast India.

Area of isolation	*In vitro* CQ sensitivity status	*Pfmdr1*- codon 86		*Pfcrt*-codon76	
		Allele-N86	Allele-86Y	Allele-K76	Allele-76T
Assam (n = 65)	CQR* (n = 50)	0	50	0	50
	CQS** (n = 15)	15	0	15	0
Arunachal Pradesh (n = 50)	CQR (n = 50)	0	50	0	50

* chloroquine resistant; ** chloroquine sensitive.

In total, 100 CQ resistant isolates (from *in vitro*) showed the mutation at codon 86Y, while in CQ sensitive isolates, wild type allele (N86) was observed. In these isolates, N86Y mutation was found to be associated with the *in vitro* CQ sensitivity status (Chi^2^ test, P<10^−5^). The K76T mutation was also found to be associated with the *in vitro* CQ susceptibility in all the isolates (Chi^2^ test, P<10^−5^). The linkage disequilibrium (LD) between the alleles of these two loci (*Pfmdr1-*N86Y and *Pfcrt*-K76T) was analyzed by calculating the LD constants D' [23] and r^2^ [24]. The LD constants show strong LD between these loci (D' = 1.00; r^2^ = 1.00) (Table 2).

**Table 2:** Linkage disequilibrium between alleles of *Pfmdr1* codon 86 (on chromosome) five and *Pfcrt* codon 76 (on chromosome seven).

	*Pfcrt-*76T	*Pfcrt-*K76	D'	r^2^	P
*Pfmdr1-*86Y	100	0	1.00	1.00	<10^−5^
*Pfmdr1-*N86	0	15			

## Discussion

Since its first report in 1950's, CQ resistance had spread worldwide [29]. In India, the first case of CQ resistance was reported in 1973 from Karbi-Anglong district in the Assam [30]. Since then, it has gradually spread West and South [11]. The fast rate of emergence of CQ resistance has become a major hurdle in the control of malaria.

The development of molecular techniques for the rapid identification of drug resistant parasites is of immense importance for the epidemiology, and information on the choice of antimalarial treatment regimens. In the present study, the *in vitro* CQ susceptibility pattern of 115 isolates was compared with that of point mutations in the genes *Pfmdr 1* and *Pfcrt.* The development of the CQ resistance phenotype is a complex and probably cumulative phenomenon where more than one gene with one or more mutation(s)/polymorphism(s) might contribute to the development of CQ resistance [4].

In the present study, the CQ sensitivity assay and DNA isolation were performed with the fresh clinical isolates of *P. falciparum*, immediately after the collection of blood. Continuous *in vitro* culture induces the selection of sub population of parasites, and hence does not truly represent parasites of the *in vivo* infection [33]. In previous studies, culture-adapted isolates or clones of mutations in *Pfmdr1*were used [20]. The CQ resistant strains become CQ sensitive on withdrawal of the drug or on continuous passages during culture [34]. Therefore, genotyping of such culture-adapted isolates would be misleading. The results of *in vitro* CQ susceptibility tests showed that out of a total 115 clinical isolates, 100 isolates were resistant towards the CQ while only 15 isolates showed sensitivity to CQ. Interestingly, 100% of the isolates from the Arunachal Pradesh and 77% isolates of the Assam showed the *in vitro* CQ resistance indicating the alarming situation of CQ resistance in *P. falciparum*.

On molecular detection of point mutations in *Pfmdr1,* a strong association was observed between codon 86Y mutation and *in vitro* CQ resistance in these isolates. These findings corroborate well with the previous findings [12], [17], [19], [32]. In the present study, all the CQ resistant isolates showed mutational change at position 86Y confirming the role of this mutation in the CQ resistance. It is likely that the 86Y allele of *Pfmdr1* is of functional relevance. A point mutation in the *Pfmdr1* gene results in a change of an amino acid at codon 86, 184, 1034, 1042 and 1246. However, contrasting observations are available in this regard. It has been observed that the Southeast Asian CQ resistant isolates have a change in the amino acid at codon 86 from asparagine to tyrosine (N86Y) [31]. While CQ resistant South American isolates have shown mutational changes at codon 184, 1034, 1042 and 1246. Out of these, the mutation at codon 86 appears to be important since it is involved in the substrate specificity of the gene product (P- glycoprotein), and hence may alter the transport activity of the protein [31], [38]. Similarly, due to mutations in *Pfcrt* gene, mutations involving the substitution from lysine (K) to threonine (T) at position 76 (K76T) has also been observed consistently in the CQ resistant strains [4], [11].

The analysis of K76T mutation in *Pfcrt* gene revealed its 100% association with the *in vitro* CQ resistance. In an earlier study, significant association (linkage disequilibrium, LD) between the alleles *Pfmdr1* 86Y and *Pfcrt* 76T was observed [12], [35]–[36]. Significant association between the *Pfmdr1* 86Y and the EC_50_ of CQ among clones with the *Pfcrt* 76T allele suggests the role of both the mutations in CQ resistance. The results of the present study corroborate with the earlier studies, which showed significant association between *Pfmdr1* N86Y and *Pfcrt* K76T [19], [23], [37].

The LD test measures the linkage between genes/alleles on different chromosomes under a restrictive set of conditions or external influence (selection under the drug pressure) [21]–[22]. To analyze the LD between alleles at codon 86 of *Pfmdr1* gene on chromosome 5 and alleles at codon 76 of *Pfcrt* gene on chromosome 7, LD constants (D' and r^2^) were calculated. A high LD between these two loci (D' = 1.00 and r^2^ = 1.00) was observed. These findings are in agreement with the findings of Adagu and Warhurst, [38], where high LD values were observed between these two alleles. The CQ has been the first line drug for treatment of uncomplicated malaria in the Assam and Arunachal Pradesh. Thus, our findings in combination with previous evidence suggested that the LD between *Pfcrt-*K76T and *Pfmdr1*-N86Y alleles might be due to the strong directional selection of the alleles by CQ drug pressure in the population of the studied area. These results further suggest that *Pfmdr1* (N86Y) and *Pfcrt* (K76T) are potentially useful markers of the assessment of *in vitro* CQ resistance in the Northeast India. These can be used for the rapid diagnosis and surveillance of CQ resistance and may have potential use in the monitoring of *in vivo* therapeutic efficacy of CQ in malaria endemic areas.
